# The self-similarity theory of high pressure torsion

**DOI:** 10.3762/bjnano.7.117

**Published:** 2016-09-07

**Authors:** Yan Beygelzimer, Roman Kulagin, Laszlo S Toth, Yulia Ivanisenko

**Affiliations:** 1 Laboratory of Excellence on Design of Alloy Metals for low-mAss Structures (DAMAS), Université de Lorraine, Île du Saulcy, Metz, F-57045, France; 2 Donetsk Institute for Physics and Engineering named after O.O. Galkin, National Academy of Sciences of Ukraine, pr. Nauki 46, Kyiv 03028, Ukraine; 3 Institute of Nanotechnology (INT), Karlsruhe Institute of Technology (KIT), Hermann-von-Helmholtz-Platz 1, Eggenstein-Leopoldshafen, 76344, Germany; 4 Laboratoire d’Etude des Microstructures et de Mécanique des Matériaux (LEM3), Université de Lorraine, UMR 7239, Metz, F-57045, France

**Keywords:** deformation mechanisms, high pressure torsion, nanocrystalline metals, self-similarity, severe plastic deformation

## Abstract

By analyzing the problem of high pressure torsion (HPT) in the rigid plastic formulation, we show that the power hardening law of plastically deformed materials leads to self-similarity of HPT, admitting a simple mathematical description of the process. The analysis shows that the main parameters of HPT are proportional to β*^q^*, with β being the angle of the anvil rotation. The meaning of the parameter *q* is: *q* = 0 for velocity and strain rate, *q* = 1 for shear strain and von Mises strain, *q* = *n* for stress, pressure and torque (*n* is the exponent of a power hardening law). We conclude that if the hardening law is a power law in a rotation interval β, self-similar regimes can emerge in HPT if the friction with the lateral wall of the die is not too high. In these intervals a simple mathematical description can be applied based on self-similarity. Outside these ranges, the plasticity problem still has to be solved for each value of β. The results obtained have important practical implications for the proper design and analysis of HPT experiments.

## Introduction

High pressure torsion (HPT) is a severe plastic deformation process, which is widely used for producing nanocrystalline metals and alloys [1–3]. The generally accepted theory of HPT is based on the assumptions of uniformity of simple shear deformation along the height of the specimen and that there is no slippage between the sample and the anvils. This theory gives a simple expression for the shear strain

[1]
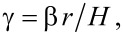


where β is the torsion angle of the anvil, *r* is the radial position and *H* is the height of the disc.

However, a number of recent experiments and numerical simulations show that the true plastic flow during HPT can differ significantly from the theoretical predictions given by the simple scheme above. In particular, in [4–5] a problem of coupled phase transformations and plastic flows under torsion at high pressure in a rotational diamond anvil cell was investigated. It has been shown that not only the stress, but the strain state of the sample are strongly dependent on the rheological properties of the material. In addition, it has been shown that the assumption of no slippage between the sample and the anvils is too simple in some cases, and leads to significant errors in the description of phase transformations. A correct approach to take into account the slippage in finite element simulations has been developed in [4–5]. A simple analytical model taking into account the slippage has been offered in [6]. It leads to an equation similar to Equation 1 for shear strain with a factor decreasing the angle of rotation of the sample due to slippage. The same result was obtained experimentally in [7].

The effect of the elasticity of the anvils on the geometry of the sample and the distribution of the shear strain has been investigated in [8–10]. In particular, it was shown in [10] that the samples are further deformed during unloading of the anvils and this produces a peak in the strain in the central region of the disc.

Finite element modeling is used in [11–12] to show variations of the stress–strain state along the height of the specimen. In particular strong shear strain localization near the contact surface between sample and anvil was quantified in [12]. In [13] a dead zone is found along the border of the specimen. A double-swirl pattern in the plane perpendicular to the torsion axis is experimentally shown in [14].

The above results point out the fact that plastic flow during HPT may have a fairly complex behavior, which should be taken into account when investigating materials obtained by this method. Thus, the following question can be raised: Under what conditions can HPT be modeled in a simple manner?

**Figure 1:**
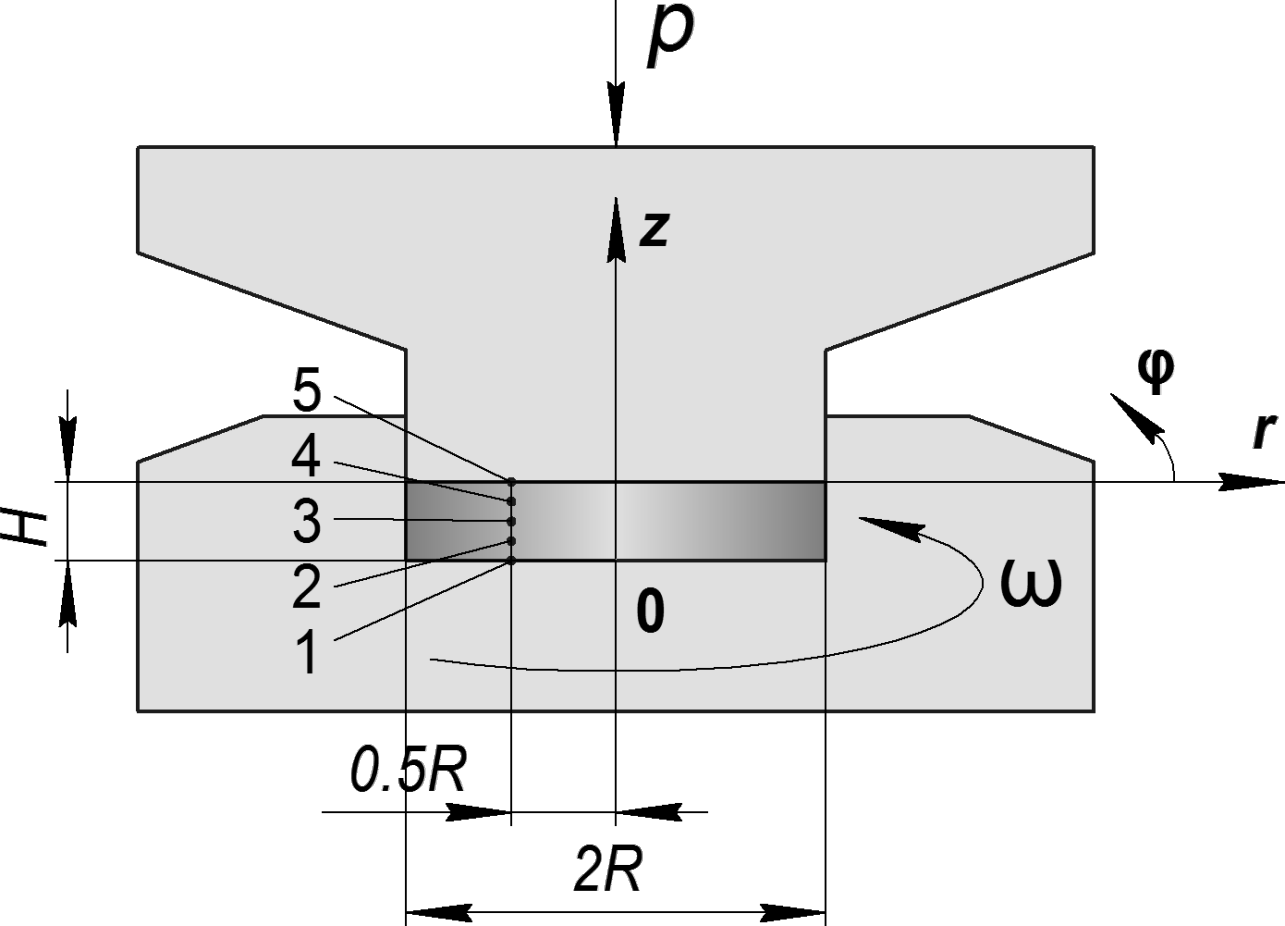
Schematic geometry of the constrained HPT process (the notation is defined in the main text).

We will call an HPT process *simple* if the shear strain in every point of the specimen is a linear function of the torsion angle of the anvil, i.e.,

[2]
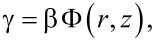


where Φ(*r*,*z*) is a differentiable function of *r* and *z*, and *z* is the coordinate along the specimen axis as shown below in Figure 1. This relationship generalizes Equation 1 by inheriting its main property: the separation between the dependency on β and the dependency on *r* and *z*. According to Equation 2, once we have Φ(*r*,*z*), we have a tool for calculating γ at every point of the specimen, for any value of the torsion angle. This is exactly what makes HPT simple. If, on the other hand, one cannot separate the β-dependence from the dependence on the coordinates, i.e., γ = Γ(*r*,*z*,β), then, in order to compute γ, one has to solve a plasticity problem for every angle β. This is what makes the process complex in terms of its mathematical description.

The question what makes a complex physical process simple to describe emerges naturally, and the answer often has to do with the self-similarity of the process [15]. Self-similarity makes a complex process simple to describe because the process just repeats itself at different scales of time or space. For example, the self-similarity of powerful blast waves has allowed G. I. Taylor to predict damage from nuclear explosions even before such damage was observed for the first time. These results were obtained at the beginning of 1941 but were declassified only in 1950 [16]. Many other examples of simple descriptions of complex processes based on their self-similarity are given in [15].

In this work we investigate plastic flow during constrained HPT. We show that, within the rigid plastic framework, the problem we are considering has a self-similar solution if the deformed material satisfies a power hardening law. In this case, the shear strain is a linear function of the torsion angle of the anvil and can be described by Equation 2. We analyze different properties of self-similar regimes of HPT, and show that the self-similarity of HPT at the macro-level is connected to the self-similarity of the structure of the material at the micro-level hypothesized in [17].

## Model

### 1 Rigid plastic flow formulation for HPT

Figure 1 schematically shows the constrained HPT process [2].

Within the rigid plastic flow framework [18], the definition of the stress–strain state (SSS) during HPT is reduced to solving a set of equations in the cylindrical coordinate system, *r*, φ, *z* (see Figure 1), including (a) the equilibrium equations obtained from div(σ) = 0:

[3]
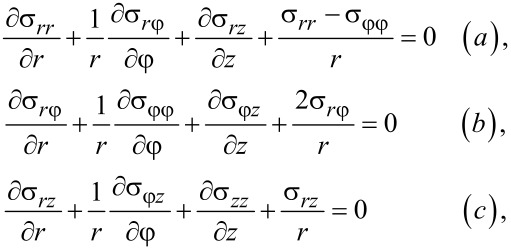


(b) the relations between the components of the strain rate tensor and the velocity vector:

[4]
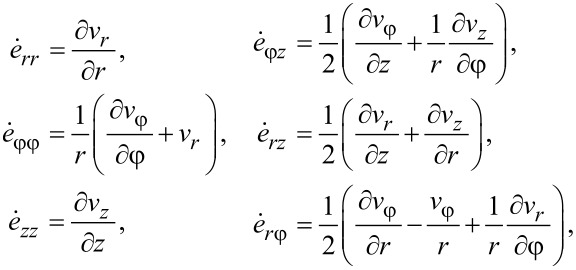


(c) the von Mises plasticity condition:

[5]
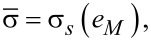


(d) the associated flow rule:

[6]
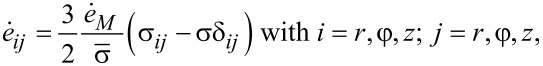


and (e) the constant volume condition:

[7]
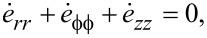


where σ*_ij_* and 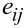 with *i* = *r*,φ,*z*; *j* = *r*,φ,*z* are the components of the stress and strain rate tensors respectively; ν*_i_* with *i* = *r*,φ,*z* are the components of the velocity vector; σ = 1/3·σ*_ij_*·δ*_ij_* is the hydrostatic stress,


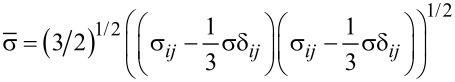


is the equivalent stress, 
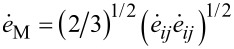
 is the von Mises strain rate, and σ*_s_*(*e*_M_) is the flow stress of the material that depends on the von Mises strain:

[8]
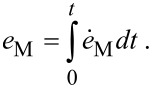


The set of Equations 3–7 is solved under the following boundary conditions:

[9]
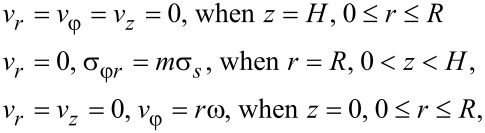


where *m* is the friction coefficient and ω is the torsion rate of the anvil.

Under the terms of Equation 9 we believe there is no slippage in the contact between the sample and the surface planes of the anvils. According to the experiments in [7] this can always be achieved by applying a sufficiently high pressure. In subsection 2, we will show that under certain conditions, the HPT problem has a self-similar solution both with and without slippage.

The torsion angle β does not explicitly appear in Equations 3–7 but the problem is time-dependent because the flow stress σ*_s_* depends on the von Mises strain *e*_M_, which in turn increases with β according to Equation 8.

The global solution of Equations 4–9 has the following form:

[10]
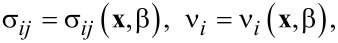


where **x** is the position vector.

### 2 A self-similar solution of the rigid plastic flow problem for HPT

Let *y* = *f*(*x*,*t*) describe the spatial distribution of a quantity *y* as a function of time *t*, where *x* is the spatial coordinate. The process is called self-similar, if the spatial distribution of *y* at any time *t* can be obtained from a reference solution at time *t*_0_ by a simple similarity transformation:

[11]
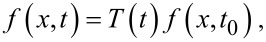


where *T*(*t*) is time-dependent.

Thus, the spatial distribution of *y* varies with time while remaining always geometrically similar to itself. This definition generalizes the concept of similarity in geometry where two figures are called similar if one can be obtained from the other by uniformly scaling along all dimensions [15].

It can be shown [15] that a self-similar process satisfies the following scaling law:

[12]
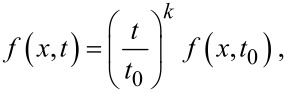


where *k* is a parameter.

This scaling law suggests that a self-similar solution to the HPT problem should have the following form:

[13]
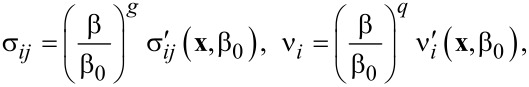


where *g* and *q* are parameters to be defined, and 
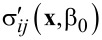
 and 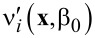 represent a solution to the problem for a given torsion angle β_0_.

In the following it will be shown that, under certain conditions, the relationships in Equation 13 satisfy the set of Equations 3–7 and the boundary conditions in Equation 9, i.e., the HPT problem has a self-similar solution.

We look for a self-similar velocity field in the following form:

[14]
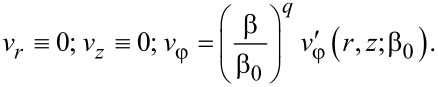


It can be readily seen that such a velocity field automatically satisfies the condition of constant volume (Equation 7) and also the boundary conditions (Equation 9) for *v_r_* and *v_z_*. According to Equation 9, when *z* = 0, the value of *v*_φ_ does not depend on β. This implies that *q* = 0, i.e., a self-similar velocity field under HPT should not depend on the torsion angle of the anvil. In this case, the components of the strain-rate tensor have the following form:

[15]
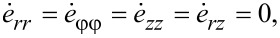


[16]
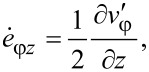


[17]
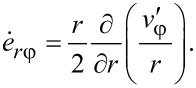


Under these conditions the von Mises strain rate is defined by:

[18]
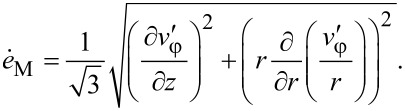


Inserting this expression into Equation 8 it can be seen that if HPT is self-similar, the von Mises strain in every point of the specimen is a linear function of the torsion angle β:

[19]
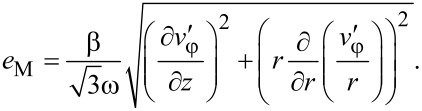


Now we determine the components of the stress tensor for self-similar flow. They should satisfy the set of equilibrium equations (Equation 3), the von Mises plasticity condition (Equation 5) and the associated flow rule (Equation 6). The latter, with Equations 15–17, gives:

[20]
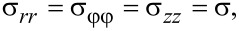


[21]
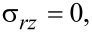


[22]
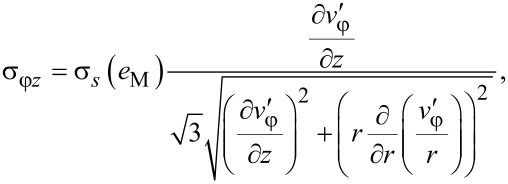


[23]
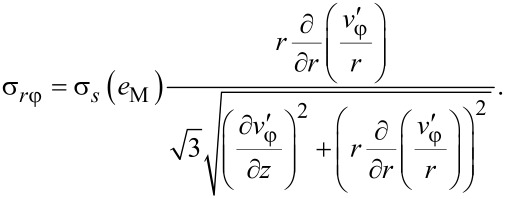


It can be readily seen that the right hand sides of Equation 22 and Equation 23 scale with β only when the material hardens according to a power law (where von Mises strain is a linear function of β in Equation 19):

[24]
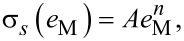


where *A* and *n* are parameters. Inserting Equation 24 and Equation 19 into Equation 22 and Equation 23, we get:

[25]
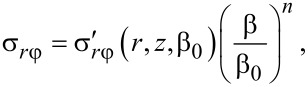


[26]
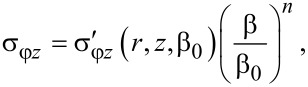


where

[27]
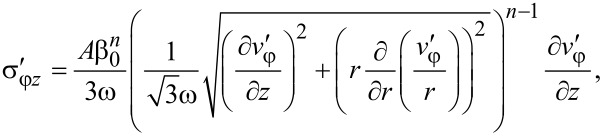


[28]
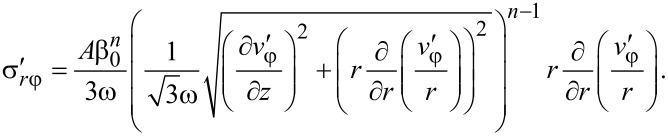


Comparing Equation 25 and Equation 26 with Equation 13, we obtain: *g* = *n*.

According to Equation 20 and Equation 21, and using the torsional symmetry (derivatives with respect to φ are 0), Equation 3a and Equation 3c have the following form:

[29]
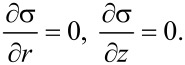


Thus, according to Equation 20, the normal stresses do not depend on the coordinates. We obtain:

[30]
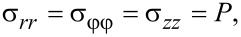


where *P* is the pressure applied in HPT.

By virtue of the torsional symmetry and using Equation 25 and Equation 26, Equation 3c becomes:

[31]
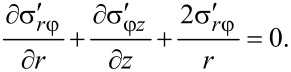


Inserting this into Equation 27 and Equation 28, and after some algebraic manipulations, a second order partial differential equation is obtained:

[32]
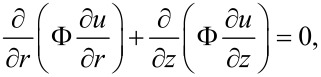


where


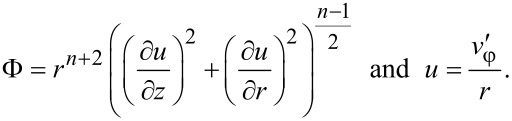


Equation 32 is a steady-state diffusion equation [19], where *u* represents the concentration, and the function Φ, which depends on the absolute value of the gradient of the concentration and on the radius *r*, serves as the diffusion coefficient. According to Equation 9, the boundary conditions for Equation 32 are the following:

[33]
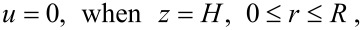


[34]
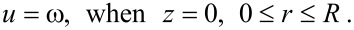


The equation gives another boundary condition for σ*_r_*_φ_ when *r* = *R*, 0 < *z* < *H* (see Equation 9). After inserting Equation 23 and doing some algebraic operations, we get:

[35]
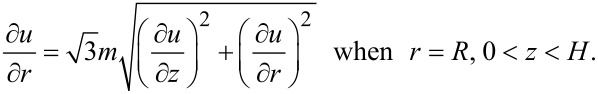


In [19] it is shown that the problem defined by Equations 32–35 has a unique solution. Thus, if the hardening law has a power form, the problem of rigid plastic flow in HPT has a self-similar solution, according to which the following relations hold for the velocity vector

[36]
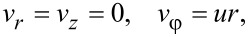


for the strain rate tensor

[37]
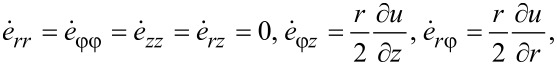


the von Mises strain

[38]
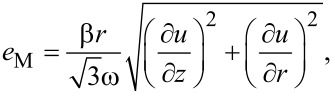


and the shear stress

[39]
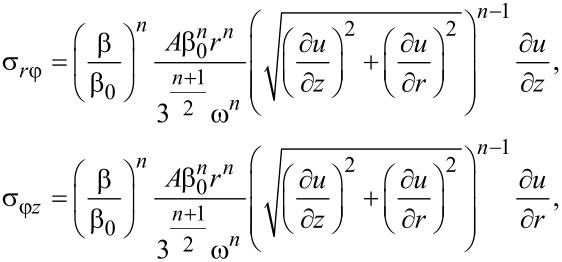


where the exponent *n* is equal to the exponent in the hardening law of the material, while the function *u* = *u*(*r*,*z*) is the solution of Equations 32–35.

It follows from Equation 39 that if the evolution of the process is self-similar, the torque *M*(β) during HPT satisfies a power law, i.e.,:

[40]
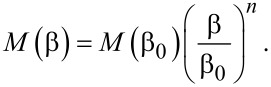


If the hardening law does not satisfy a power law form, there is no self-similar solution.

When there is no friction on the cylindrical surface of the anvil (*m* = 0) a solution of Equations 32–35 can be readily obtained for an ideally plastic material (*n* = 0). This case is of interest because due to saturation in strain hardening, metals are perfectly plastic in the high strain regime [20–21]. One can verify by direct substitution that when *n* = 0 and *m* = 0, the function u = [(*H* – *z*)/*H*]·ω satisfies the differential Equation 32 and the boundary conditions (Equations 33–35). In this case, the sample is deformed by a simple shear pattern and the fields of velocity, strain rate and stress do not depend on the rotation angle. Furthermore, Equation 38 for the von Mises strain simplifies to the well-known formula


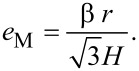


The simple theoretical scheme based on simple shear is a special case of a self-similar solution of the HPT process. Please note that simple shear can only be homogeneous along the height of the specimen. If this is not the case, then 
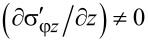
 and Equation 31 would imply that 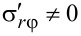 (to satisfy the equilibrium Equation 31), consequently, for any shear in the plane φ*z* there is always some shear in the plane φ*r*.

We note that all the arguments in this section are formally valid not only when ω = const, but also when this parameter is a function of the radius. It can then be represented in the form ω(*r*) = *k*(*r*)ω. One can interpret ω(*r*) as an angular velocity of the metal on the surface plane of the anvil. If *k* is equal to 1 there is no slippage. If *k* is smaller than 1 the possibility of slippage is taken into account. Hence, *k* can be called “slippage coefficient”. Thus, all conclusions about the self-similarity of the HPT process made above are valid when the slippage coefficient is constant or a given function of *r* only.

We can conclude this section with the following interesting observation. It has been shown in [22] that the power-law hardening is originating from the self-similarity of the microstructure, while it has been shown in the present work that the self-similarity of HPT at the macro-level requires power-law hardening, Thus, there is a relationship between self-similarities at different scales in HPT: Self-similarity at the micro-level is a physical cause of self-similarity at the macro-level.

### Results of numerical simulations

In subsection 2 of the section “Model” it has been shown that if the hardening law of the material has a power-law form, the HPT process must evolve in a self-similar fashion, and its parameters must have a power dependence on the torsion angle of the anvil (satisfying the scaling law). For the velocity field and the strain-rate field, the exponent is 0 in this case (the right hand sides of Equation 36 and Equation 37 do not depend on β). For shear strain and for von Mises strain, the exponent is 1 (see Equation 38). Finally, for shear stress and torque, the exponent is equal to the exponent of the hardening law (see Equation 39 and Equation 40).

The second-order differential Equation 32 cannot be solved analytically. Therefore, numerical simulations are needed to identify self-similar solutions. In this section, we illustrate this conclusion by finding a numerical solution to a concrete HPT problem using the finite element method. Moreover, we will use numerical modeling to show that whenever a given hardening curve has an interval of power dependence, the process will enter a self-similar regime. The regime persists for some time (for an interval of rotation) and then disappears.

**Figure 2:**
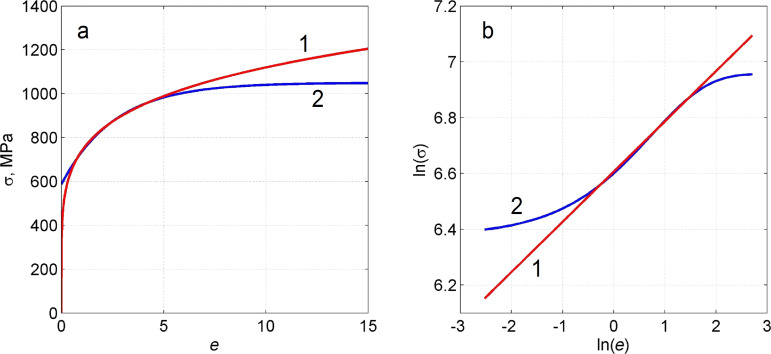
Strain hardening curves used in the numerical simulations. (a): Curve 2 is the experimentally measured stress–strain curve [24] and curve 1 is a fit by the power function σ [MPa] = 740*e*^0.18^. (b): log–log Plot of (a) showing that curve 1 is a good approximation of curve 2 in some interval.

All calculations were done using the DEFORM software employing a 2D axisymmetric torsion model [23]. The number of initial meshes for the workpiece was 10,000, which was sufficient to explore the local deformation behavior. There was no need to update the mesh because it remained undistorted during the simulation. The process is described in Figure 1. The geometric parameters were *H* = 3 mm and *R* = 10 mm. All calculations were done assuming the rigid plastic flow model with strain hardening curves displayed in Figure 2. Curve 1 is experimentally measured [24] for compacted iron powder. Curve 2 is a power function σ [MPa] = 740*e*^0.18^, providing a good fit to Curve 1 in the interval between 0.5 to 5.0, see also in the log–log plot (Figure 2b).

**Figure 3:**
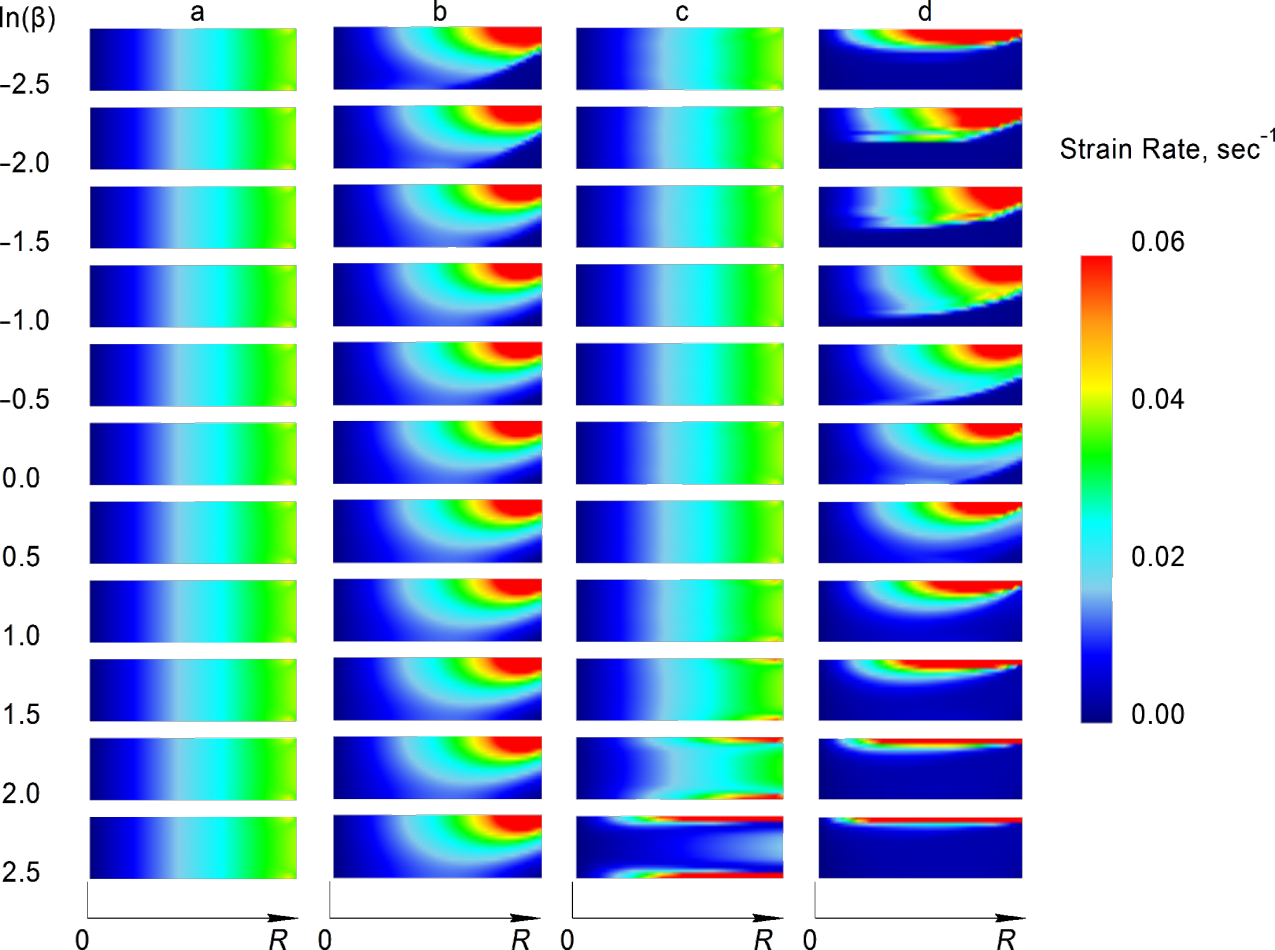
Maps of the von Mises strain rate in the (*r*,*z*) plane for different rotation angles β. An index above each figure corresponds to the index of the respective case in Table 1.

**Figure 4:**
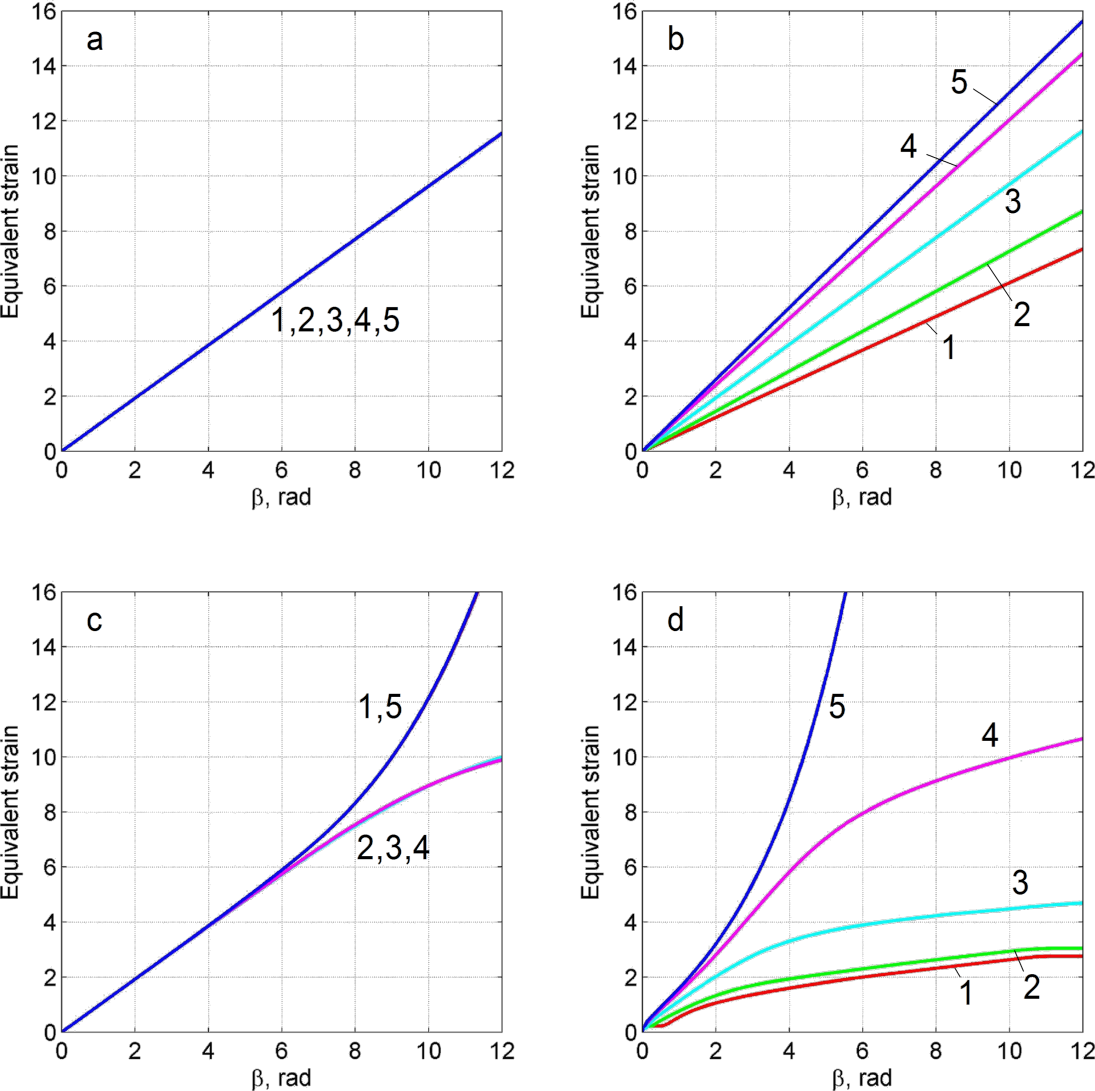
Dependence of the von Mises strain on β in points 1–5 shown in Figure 1. The indices a, b, c and d correspond to the simulation conditions shown in Table 1.

**Figure 5:**
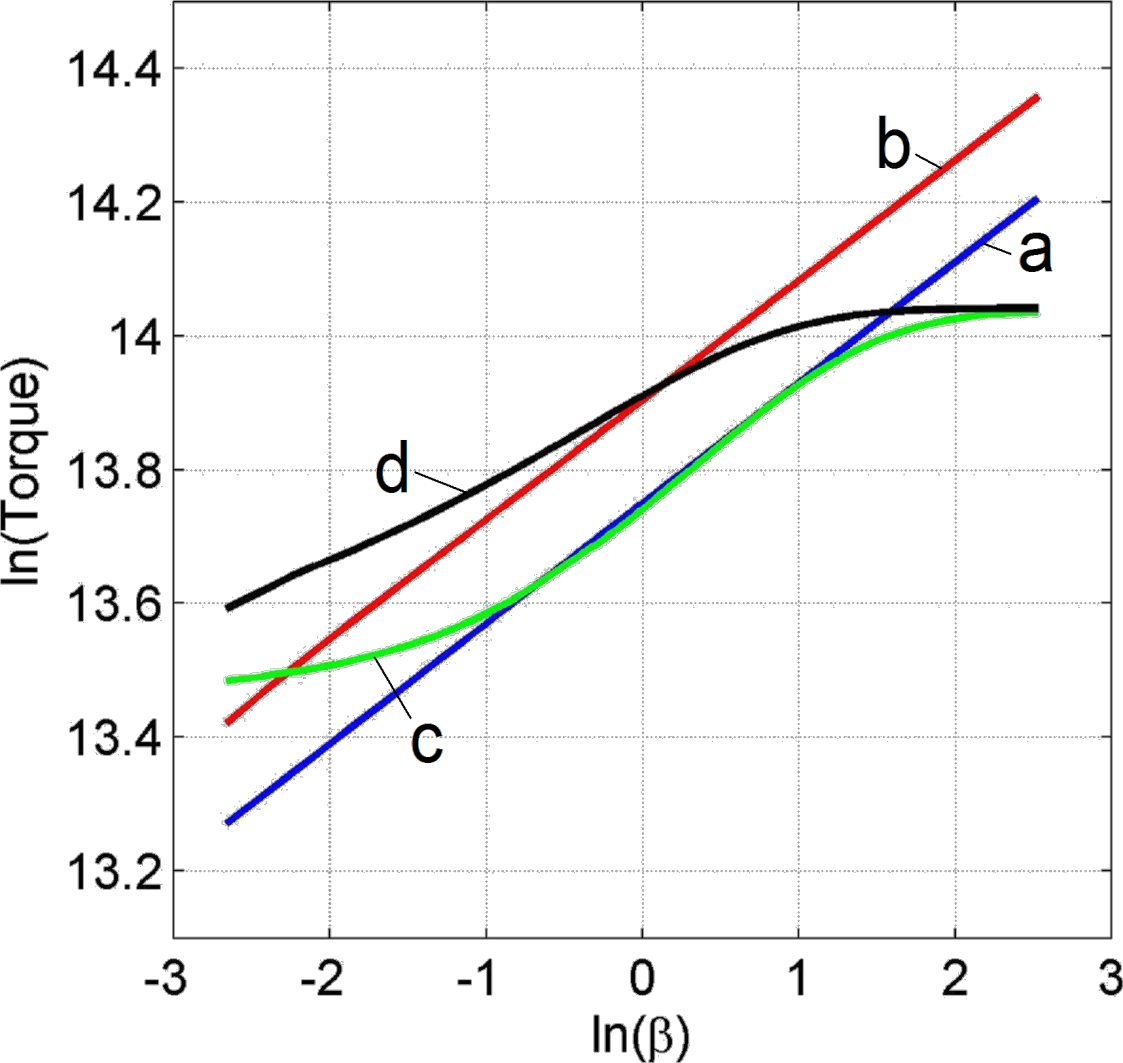
Dependence of torque on β in log–log coordinates. Each curve is indexed with the index of the respective conditions given in Table 1.

We considered two values for the friction coefficient *m* on the side surface of the bottom anvil: *m* = 0.25 and *m* = 0. The first case simulates a high-friction case while the second corresponds to low friction on the side surface. A description of our numerical simulation conditions are given in Table 1. The results of the simulations are displayed in Figures 3–5.

**Table 1:** Conditions of the numerical simulations.

simulation condition	a	b	c	d

hardening curve	power law (curve 1)	power law (curve 1)	no power law (curve 2)	no power law (curve 2)
friction, *m*	0	0.25	0	0.25

### Analysis of the simulation results obtained by power-law hardening (cases a and b)

These cases revealed self-similar regimes for HPT, in complete accord with the results of the analysis presented in subsection 2 of section “Model”. Indeed, as can be seen in Figure 3a,b, the strain-rate field does not change as the torsion angle increases. This implies that the strain depends linearly on β, at any point of the specimen. This linear dependence is illustrated in Figure 4a,b for five characteristic points shown in Figure 1. The plots in Figure 5 show that the logarithm of the torque depends linearly on the logarithm of the β. This means that the torque is a power function of β. The exponent in both (a) and (b) is the respective exponent of the hardening law: *n* = 0.18.

The numerical calculations are in accordance with the analysis of subsection 2 of section “Model”: Self-similarity is caused precisely by a power law form of the hardening curve, while friction at the side surface of the specimen affects only the dependence of the stress–strain state on the spatial coordinates. If there is no friction (regime a), the stress–strain state is uniform along the height of the specimen.

### Analysis of the results of the numerical simulations using a hardening curve with an interval of power-law dependence (cases c and d)

In the case of low friction (*m* = 0), the strain-rate field is constant in some interval of β (Figure 3c). The plot of ln *M* as a function of ln β coincides with the straight line in the same interval (see Figure 5c). The exponent in this straight line is the respective exponent in the hardening law, namely *n* = 0.18. Thus, HPT has a self-similar behavior within this interval of β. If the friction coefficient is large (*m* = 0.25), the interval of self-similarity is practically nonexistent (see Figure 3c, Figure 4c and Figure 5c).

Based on the simulation results, we can conclude that when the hardening law has a power law interval, self-similar regimes can emerge in HPT in some ranges of β when side-wall friction is small. This intermediate self-similarity guarantees a simple mathematical description of the process in the corresponding β range. Outside this range, the plasticity problem has to be solved for each value of β, since the simple power-law function dependencies are not valid outside the interval. In particular, in order to calculate the shear strain, we cannot use the linear dependencies of Equation 1 and Equation 2, which, according to Figure 4c,d, give results that are substantially different from the results obtained numerically. In the absence of self-similarity, the strain value depends on the hardening curve.

## Discussion

We have shown in subsection 2 of the section “Model” that the uniform simple shear state along the height of the specimen is a solution of the HPT problem in the case of ideally plastic material when there is no friction on the cylindrical surface of the anvil. On the other hand, according to Figure 3c the saturation in hardening in HPT (see curve 2 in Figure 2) leads to a localization of the deformation in two thin layers near the plain surfaces of the anvils. One can verify by direct substitution that a velocity field in the form of two thin layers of simple shear separated by a cylindrical rigid zone is also a solution of the HPT problem for a perfectly plastic body when *m* = 0. This is a known problem of non-uniqueness of solutions of the problem of the ideal rigid plastic body [25]. A well-known method for the regularization of this problem is introducing viscosity [25] that allows for the allocation of only one velocity field. In the case of large deformation of metals under HPT, such regularization has a physical justification. It is due to the fact that the flow stress of metallic materials in a sub-microcrystalline state significantly depends on the strain rate, even at room temperature. For this reason, there is need in the future to find self-similar regimes of HPT in the frame of a viscoplastic model.

A natural question arises: What do these results imply for the practice of HPT processing? This paper shows that, under certain conditions, HPT evolves in a self-similar way, admitting a simple mathematical description. This means that for some range of torsion angles (henceforth, scaling range), the HPT parameters are proportional to (β/β_0_)*^q^*, where β_0_ is an arbitrary angle that belongs to the scaling range (e.g., its median), *q* = 0 for velocity and strain rate, *q* = 1 for shear strain and for von Mises strain, *q* = *n* for shear stress and torque (where *n* is the exponent of the power-law function approximating the hardening curve in the given interval).

To apply this result to HPT in practice, let us formulate the following scaling hypothesis: If, experimentally, there is a range of torsion angles where the torque is a power function of the angle, then the process is self-similar in this range. We give a concrete example illustrating the application of this hypothesis.

**Figure 6:**
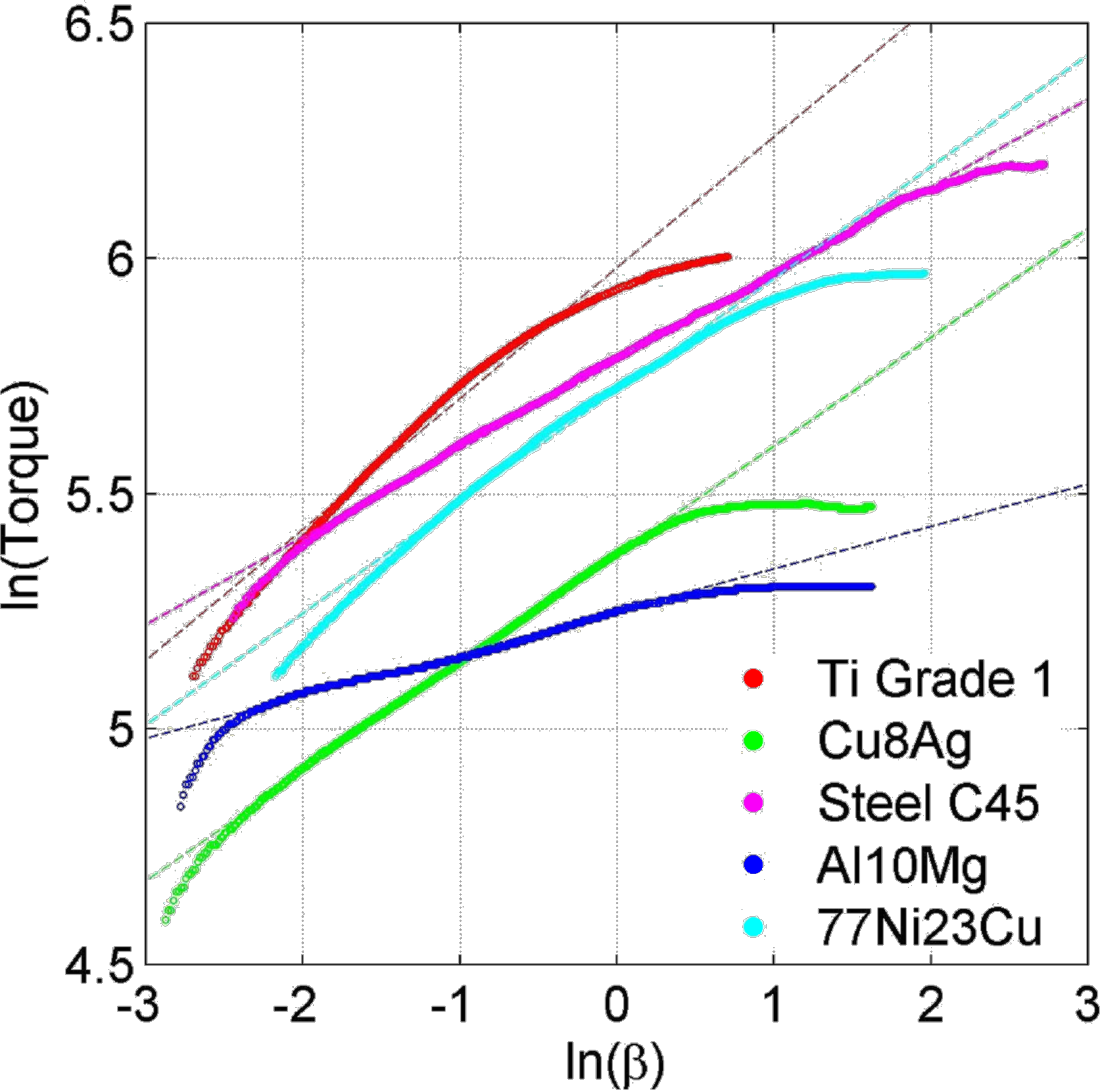
Log–log plot of the dependence of the torque on β, obtained from experimental data.

We have performed HPT experiments with different materials. We used anvils of different types together with different sample sizes. The experimental conditions are presented in Table 2. Figure 6 shows a log–log plot of the dependence of the torque on β, for different alloys.

**Table 2:** The conditions of the HPT experiments.

	1	2	3	4	5

material	Ti Grade 1	Cu8Ag	Steel C45	Al10Mg	77Ni23Cu
anvil type	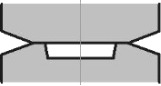	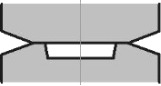	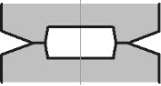	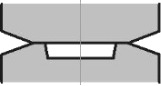	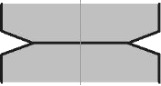
specimen size	Ø10 × 0.2 mm	Ø10 × 0.2 mm	Ø8 × 0.4 mm	Ø10 × 0.2 mm	Ø12 × 0.15 mm
pressure, GPa	7	4.5	6	5	7
rotation, rpm	1	1	1	1	1
temperature, °C	20	20	380	20	20

In all plots in Figure 6, there are clear linear intervals testifying that at some stage of the process, the dependence of the moment on β has a power form. Table 3 gives scaling ranges of beta and their respective exponents.

**Table 3:** Scaling ranges and their respective exponents.

		Ti Grade 1	Cu8Ag	Steel C45	Al10Mg	77Ni23Cu

*n*		0.278	0.231	0.186	0.09	0.273
self-similarity starts	rad	0.18	0.09	0.15	0.10	0.30
	turns	0.028	0.015	0.024	0.016	0.048
self-similarity stops	rad	0.72	1.34	8.00	1.65	1.60
	turns	0.11	0.21	1.27	0.26	0.25

In the scaling ranges in Table 2, the slope *n* of the tangent line is equal to the exponent of the hardening curve, while the shear strain in any point of the specimen is given by the formula:

[41]
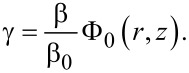


Here Φ(*r*,*z*) is the shear strain distribution in the specimen when β = β_0_.

## Conclusion

This paper shows that HPT admits a simple mathematical description when the deformed material hardens according to a power function. In this case, the process is self-similar, and all its parameters have a power form dependence on the torsion angle β of the anvil. In particular, the shear strain in any point of the specimen is a linear function of the torsion angle β.

When the hardening curve has an interval that is well approximated by a power function, HPT can become self-similar in the corresponding range of β. According to the scaling hypothesis formulated in the Discussion section, this range corresponds to the interval where the plot of ln *M* as a function of ln β can be well approximated by a straight line.

To conclude, any simple scaling expressions for HPT parameters in terms of β are valid only in the scaling ranges. Outside the scaling ranges, we still have to solve a plasticity problem to determine the HPT parameters for each β value. In particular, one cannot use Equation 1 and Equation 2 to compute shear strain in this case.

In [22] the scaling nature of the power-law interval of the hardening curve was established based on the self-similarity of the microstructure. Therefore, self-similarity at the micro-level is a physical reason of self-similarity at the macro-level.
